# Distribution of Alpha Thalassaemia Gene Variants in Diverse Ethnic Populations in Malaysia: Data from the Institute for Medical Research

**DOI:** 10.3390/ijms140918599

**Published:** 2013-09-10

**Authors:** Rahimah Ahmad, Mohamed Saleem, Nisha Sabrina Aloysious, Punithawathy Yelumalai, Nurul Mohamed, Syahzuwan Hassan

**Affiliations:** 1 Haematology Unit, Institute for Medical Research, Jalan Pahang, 50588 Kuala Lumpur, Malaysia; E-Mails: nisha@imr.gov.my (N.S.A.); punithawathy@imr.gov.my (P.Y.); edayat_qber@hotmail.com (N.M.); syah606@yahoo.com (S.H.); 2 Genetic Research Group, Department of Biomedical Sciences, Faculty of Medicine and Health Sciences, Universiti Putra Malaysia, Selangor 43400, Malaysia; E-Mail: m_salym@hotmail.com

**Keywords:** alpha thalassaemia, Hb Adana, Malaysia, Sabah, Sarawak, Orang Asli

## Abstract

Alpha thalassaemia is highly prevalent in the plural society of Malaysia and is a public health problem. Haematological and molecular data from 5016 unrelated patients referred from various hospitals to the Institute for Medical Research for *α* thalassaemia screening from 2007 to 2010 were retrieved. The aims of this retrospective analysis were to describe the distribution of various alpha thalassaemia alleles in different ethnic groups, along with their genotypic interactions, and to illustrate the haematological changes associated with each phenotype. Amongst the patients, 51.2% (*n* = 2567) were diagnosed with *α* thalassaemia. Of the 13 *α* thalassaemia determinants screened, eight different deletions and mutations were demonstrated: three double gene deletions, – – ^SEA^, – – ^THAI^, ––^FIL^; two single-gene deletions, *α*–^3.7^ and – *α*^4.2^; and three non-deletion mutations, Cd59G > A (haemoglobin [Hb] Adana), Cd125T > C (Hb Quong Sze) and Cd142 (Hb Constant Spring). A high incidence of *α*–^3.7^ deletion was observed in Malays, Indians, Sabahans, Sarawakians and Orang Asli people. However, the – – ^SEA^ deletion was the most common cause of alpha thalassaemia in Chinese, followed by the *α*–^3.7^ deletion. As many as 27 genotypic interactions showed 1023 *α* thalassaemia silent carriers, 196 homozygous *α*^+^ thalassaemia traits, 973 heterozygous *α*^0^ thalassaemia carriers and 375 patients with Hb H disease. Statistical analysis showed a significant difference in the distribution of *α* thalassaemia determinants amongst the various ethnic groups. Hence, the heterogeneous distribution of common determinants indicated that the introduction of an ethnicity-targeted hierarchical *α* thalassaemia screening approach in this multi-ethnic Malaysian population would be effective.

## 1. Introduction

Alpha thalassaemia is an inherited autosomal recessive disorder caused by a complete absence or decrease in the production of alpha globin peptides due to a deletion or mutation of one or more of the four alpha globin genes—two on each copy of chromosome 16. The normal haplotype is indicated as *αα* and its genotype as *αα*/*αα*. Deletion of one or both *α* globin genes, expressed as (–*α*/) and (– – ) respectively, represents the most common forms of *α* thalassaemia. Rarer nondeletional –*α* thalassaemia resulting from point mutations in either the *α*2 (*α*^T^*α*) or *α*1 (*αα*^T^) gene is commoner in Southeast Asia than any other part of the world. Clinically, these deletional and nondeletional forms of alpha thalassaemia can be broadly classified into four syndromes depending on the number of functional *α* globin gene inherited. When all four genes are deleted or dysfunctional, the resulting syndrome is known as haemoglobin (Hb) Bart’s hydrops foetalis or *α* thalassaemia major (– – /– – ) and is incompatible with extra-uterine life [1]. Inheritance of just one functional *α* globin gene out of the four (– – /– *α*) results in Hb H disease, which is also known as *α* thalassaemia intermedia. This is usually the result of the compound heterozygous state for *α*^0^ thalassaemia and *α*^+^ thalassaemia. Patients with this disorder show chronic haemolytic anaemia of variable severity. Hb H due to nondeletional types is relatively more severe in clinical presentation than with deletional forms [2]. The *α* thalassaemia trait (also known as *α*-thalassaemia minor), characterised by *cis* or *trans* positional loss of two *α* genes, results in heterozygosity for *α*^0^ thalassaemia (– – /*αα*) and homozygosity for *α*^+^ thalassaemia (– *α*/– *α*). The affected individuals are essentially asymptomatic with minimal hypochromic microcytic anaemia. Alpha thalassaemia silent carriers are inherited with three functional genes (–*α*/*αα*) and exhibit no clinical abnormalities; they may show normal to mild changes in the haematological indices.

Malaysia and its neighbouring Southeast Asian countries have a high prevalence of alpha thalassaemia. The gene frequencies in northern and southern Thailand were reported to be 30% and 16%, respectively [3]; 5% on the islands of Philippines [4]; 2.6%–3.2% and 2.7%–11% for *α*^0^ and *α*^+^ carriers, respectively, in Indonesia [5]; and 4.3% was expected in Brunei, where 65% of the population was Malay and 20% Chinese [6]. As in many Asian countries, *α* thalassaemia is a public health concern in Malaysia, with a gene frequency of 4.1% [7].

Malaysia has a population of 28.3 million people, with Malays and other Bumiputera groups making up 67.4% of the population. Chinese, Indians and other ethnic groups form 24.6%, 7.3% and 0.7% of the population, respectively [8]. East Malaysia, comprising Sabah and Sarawak, is populated with multiple clades of sub-ethnic groups. Aboriginal people known as Orang Asli are present in the east and peninsular Malaysia.

Population studies have indicated that the types and frequencies of the different *α* thalassaemia defects vary among different ethnic communities and tend to be geographically specific [9]. Few studies describing the frequencies of deletional and nondeletional *α* thalassaemia in Malaysia have been conducted [10], and they lack the scale and magnitude presented here; in this study, a large cohort of multi-ethnic patients was included, thereby maximising the demographic characteristics.

The present study aims to provide data on the incidence of the various forms of *α* globin gene deletions, mutations and their genotypes reflected in the diverse ethnic population of Malaysia in the context of their laboratory presentations, and to examine the data to elucidate the possibility of an ethnicity-based alpha thalassaemia screening programme.

## 2. Results and Discussion

The Malaysian meta-population at large is not homogeneous [8]. It is composed of many subpopulations partly isolated by their mate choices, which are principally based on mutually shared phenotypic characteristics. Major demes include ethnic Malays, Chinese and Indians. Malaysian Chinese are descendants of ancestral immigrants from South China, while the majority of Malaysian Indians are originally immigrants descending from Southern India. In the east, isolated from Peninsular Malaysia, lies Sabah and Sarawak, with the two major subpopulations of Sabahans and Sarawakians, respectively.

Haematological and molecular data from the patients who were referred to the Institute for Medical Research (IMR) for *α* thalassaemia screening over four years ending in 2010 were retrospectively reviewed to determine the ethnic distribution of various *α* thalassaemia determinants. The study subjects included 3139 Malays; 706 Malaysian Chinese; 258 Malaysian Indians; 391 and 41 indigenous citizenries of Sabah and Sarawak, respectively; 26 Orang Asli (aboriginal people); and 428 patients with undefined ethnic background. The ethnic communities in Sabah from which we received blood samples were KadazanDusun, Bajau, Murut, Rugus, Sungai, Suluk, Dusun, Bisaya and other citizenries. In Sarawak, the ethnic communities of Melanau, Bidayuh, Kedayan and Iban were the major contributors to the 41 blood samples received.

The most common reason for referral was hypochromic microcytic anaemia amongst patients attending general or specialist practices. Others included family members with *α* thalassaemia, members of ethnic groups at risk, children of couples at risk, partners of *α*^0^ thalassaemia carriers, negative for β thalassaemia screening and voluntary requests.

This analysis had a few significant limitations. Most importantly, as this was an audit of molecular diagnosis made on nationwide hospital-based blood samples submitted for alpha thalassaemia screening, the incidences reported here would not accurately correspond to the population prevalence of the *α* thalassaemia variant. Especially, the incidence of *α* thalassaemia due to single deletion may be under represented as their red cell indices are generally unremarkable on a routine blood counts and hence are not likely to be sent for screening. Second, the referred samples were not genotyped for β thalassaemia or other haemoglobinopathies. These observations would have determined the co-inheritance or explained the *α* thalassaemia free hypochromic microcytic anaemia. Third, as the samples were submitted only for *α* thalassaemia screening, other causes of microcytic anaemia such as iron deficiency were not determined. Finally, patients negative for the 13 *α* thalassaemia determinant screening panel were assumed to be probable *αα*/*αα* genotype without further investigation for other possible genotypes.

### 2.1. Ethnic Distribution of α Thalassaemia Determinants

Table 1 shows the molecular characterisation of alpha-globin genes in 10,032 chromosomes screened for *α* thalassaemia. Out of the 13 *α* thalassaemia determinants screened, eight variants were positive on 3138 chromosomes. These were three double gene deletions, – – ^SEA^, – – ^THAI^, – – ^FIL^; two single-gene deletions, *α*–^3.7^ and –*α*^4.2^; and three nondeletion mutations, specifically Cd59G > A (Hb Adana), Cd125T > C (Hb Quong Sze) and Cd142 (Hb Constant Spring). All of the chromosomes were negative for – – ^MED^, –(*α*)^20.5^, init ATG→A-G (Hb Vietnam), Cd30 (-3bp) and Cd35T > C (Hb Evora). In this analysis, 85% of the alpha thalassaemia chromosomes had deletional type and the remaining 15% had nondeletional mutations.

A total of 2449 (48.8%) subjects were negative for the 13 *α* thalassaemia determinants tested at the institute and were assumed to be *αα*/*αα* genotype (Table 2). More than half (*n* = 2567, 51.2%) of the patients studied were diagnosed with *α* thalassaemia, specifically 1568 Malays, 486 Chinese, 68 Indians, 226 Sabahans, 19 Sarawakians, 14 Orang Asli patients and 186 patients of unspecified ethnicity.

#### 2.1.1. *α*–^3.7^ Rightward Deletion

The highest incidence of *α*–^3.7^ was observed in Sabahans, while it was the most predominant in all ethnic groups except for Malaysian Chinese. This distinction in allele frequency observed amongst ethnic Chinese consolidates our previous finding [7] and is in accordance with the findings of other similar studies on the local population [11]. In general, the *α*–^3.7^ single gene deletion has a global distribution among all ethnic groups, and is especially prevalent in most tropical and subtropical populations studied [12].

#### 2.1.2. – – ^SEA^ Deletion

Contrary to all the other ethnic groups, the most common alpha thalassaemia determinant amongst the Malaysian Chinese was the – – ^SEA^ deletion. More than a third (75.8%) of all the alpha thalassaemia chromosomes examined amongst the Chinese ethnic group had the – – ^SEA^ deletion—which represented 29.9% of all the chromosomes studied within the entire ethnic group. Interestingly, almost all (360/364, 98.9%) *α*^0^ thalassaemia carriers in them were the result of the *αα*/– – ^SEA^ genotype. This finding is in accordance with a previous study, where a preponderance of – – ^SEA^ deletions was reported in Malaysian Chinese in a cohort of 650 pregnant women represented by the three major subpopulations of Malaysia—Malays, Chinese and Indians [10]. This increased predominance of the ––^SEA^ deletion in Malaysian Chinese elevated their predisposition of conceiving foetuses with Hb Bart’s hydrops foetalis with the – – ^SEA^/– – ^SEA^ genotype to a relatively greater extent than any other subpopulation from Malaysia. It was shown in our previous survey that at least 63 pregnancies annually were at risk of hydrops foetalis in the Malaysian population [7].

This double deletion was noted as the second most common in the Malay, Indian, Sabahan and Orang Asli groups, with an incidence of 30.2%, 9.3%, 21.5% and 26.3% respectively. The – – ^SEA^ deletion, which removes nearly 20 kb DNA that extends from the 3′ end of the *HBZps* gene through the *HbA1* gene, has been observed at high frequencies in several Southeast Asian populations and in Asians worldwide [1]. More specifically, a high prevalence of *α*^0^ thalassaemia (82.87% of all *α* thalassaemias) due to – – ^SEA^/*αα* was reported in South China [13]. Hence, the high incidence of – – ^SEA^ deletions observed in Malaysian Chinese could safely be attributed to the high gene frequency in South China. Historically, most of the Malaysian Chinese are ancestral descendants from South China [14].

#### 2.1.3. *α*^CS^*α* Mutation

Allele *α*^CS^*α*, similar to many other studies, was the most common nondeletional *α* thalassaemia mutation observed in this analysis, and was the third most frequent *α* thalassaemia determinant in Malays and Sabahans. Interestingly, the highest incidence was recorded in Orang Aslis aboriginal people (11.5%). The lowest (0.7%) was observed in Malaysian Chinese, while the mutation was not detected in Malaysian Indians or Sarawakians.

#### 2.1.4. –*α*^4.2^ Leftward Deletion

The leftward deletion –*α*^4.2^, with a uniformly low incidence of 1.1% on average, was observed in Malays, Chinese and Indians. Its incidences showed no significant difference amongst these ethnic groups (Pearson Chi-square, *p* = 0.792). The same result was reported in our previous paper in terms of 16 years old Malaysian school children, who showed a very low frequency of –*α*^4.2^ [7]. Appreciable amounts of this deletion were observed in Hong Kong and in the western fringes of Remote Oceania on haplotype backgrounds different from Southeast Asian type 1a [15].

#### 2.1.5. – – ^THAI^ Double Gene Deletion

The – – ^THAI^ double gene deletion, observed only in the three major ethnic groups of Malaysian Malays, Chinese and Indians at a uniformly low incidence (of 0.3% in average), showed no significant difference in their distribution (Pearson Chi-square, *p* = 0.574). This alelle, common in Thai population [1], may likely be lost from the Malaysian population through the action of genetic drift if the selection was not acting. The – – ^THAI^ allele observed on a single Indian chromosome was presumably due to intermarriage between the different ethnic groups.

#### 2.1.6. – – ^FIL^ Double Gene Deletion

In the Sabah subpopulation, 2.7% (6/226) of those diagnosed with alpha thalassaemia inherited the Filipino deletion. Out of these six patients, four were from the Bajau tribe and presented with genotypes *αα*/– – ^FIL^ and *α*–^3.7^/– – ^FIL^, two cases each; and two were from the Suluk tribal community, with genotypes *αα*/– – ^FIL^ and *α*^CS^*α*/– – ^FIL^. Kadazandusun, the major ethnic group in Sabah, represented in this analysis by 82 patients, did not show a single alpha Filipino deletion. This is in accordance with a previous study done in the Kadazandusun community, where 12.8% of the Kadazandusuns were found to have inherited the Filipino β thalassaemia deletion but not the alpha Filipino deletion [16].

Interestingly, all 10 patients with the Filipino deletion observed in Sarawakians were of Iban ancestry; seven had heterozygous *α*^0^ thalassaemia (*αα*/– – ^FIL^) and the other three had Hb H disease (*α*–^3.7^/– – ^FIL^).

The high incidence of the – – ^FIL^ gene in the two eastern states of Malaysia, Sabah and Sarawak could be the result of gene flow from high gene frequency neighbours. At least 2% of individuals in Philippines are carriers for the – – ^FIL^ deletion [4], and globally, the deletion is most frequent in Philippinians [1]. Therefore, resettling Philippinians could be the biggest contributor of the gene, as Philippines—with its geographical proximity to East Malaysia—shares a maritime border and was the preferred destination for a fair number of asylum seekers [17].

The single – – ^FIL^ allele observed on a lone Indian chromosome presumably represents an isolated importation of allele through intermarriages.

#### 2.1.7. Haemoglobin Adana

The nondeletional allele Cd59, (Hb Adana), observed at an incidence of 0.14% in Chinese, 1.7% in Malays, 1.9% in Orang Asli aboriginal people and 3.7% in Sarawakians, was reportedly encountered at a higher frequency of 16% in Indonesia [18]. This cline suggests that Hb Adana in Malaysian ethnic groups may be the result of gene flow from Indonesia, as there has been regular travel since antiquity between the coastal port cities of Sumatra and the Malay Peninsula. This hypothesis is further supported by the interconnected past history and the common Malayo-Polynesian language. As countries in Southeast Asia had regular contact with the Mediterranean through the silk route, this allele found in Indonesia, Malaysia and countries bordering the Mediterranean Sea might have a single origin. Hb Adana was first described in 1993 in Turkey on *α*1 and later on *α*2 in an Albanian family in 1999 [19,20].

#### 2.1.8. Cd125T > C (Hb Quong Sze)

The *α*^QZ^*α* was a private allele observed only in the Malay and Chinese ethnic groups at low incidence. This unstable *α*^+^ mutation (HBA2 c.377T > C), more frequent in Southern China, was seven times more prevalent in Malaysian Chinese than that observed for Malays. This relative increase in the Malaysian Chinese represents an ancestral gene flow and probably has not had sufficient evolutionary time to disperse to any greater degree.

### 2.2. Inter Ethnic Diversity and Screening Strategy

Although all eight determinants showed heterogeneous distribution, without clear restriction to any distinct ethnic group, inter-ethnic comparisons of their incidences showed a significant difference (Pearson Chi-square, *p* < 0.001) for all except –*α*^4.2^ and – – ^THAI^. These were probably because the three most commonly encountered *α* thalassaemia determinants demonstrated clear ethnic preponderances: *α*–^3.7^, – – ^SEA^ and *α*^CS^*α* were three times more common in Sabahans, Chinese and Orang Aslis respectively. Furthermore, the less frequent – – ^THAI^ allele observed in Chinese, and excess-of-rare allele such as ––^FIL^ observed amongst Sabahans may have complemented to this inter-ethnic differences.

Noticeably, Malaysian Malays—who exhibited all eight determinants—were the most diverse of the patient groups, while Sarawakians and Orang Aslis were the least, with only four alleles. Despite the plethora of corresponding genotypes observed in Malays, they had a close resemblance in the genotype frequencies with that observed in the Sabah, Sarawak and Orang Asli groups, as no significant differences were calculated (Mann-Whitney *U* test, *p* > 0.05; Table 3). However, the Chinese sample showed a significant difference (*p* < 0.001) in genotype frequencies with Malays, Indians and sub-clades of Sabah (Table 3). This difference could be attributable to the relative abundance of the *α* thalassaemia gene (39.5%) observed amongst Malaysian Chinese which has led to the highest incidence of alpha thalassaemia (68.8%) within the ethnic group (Table 1), and to the relative abundance of – – ^SEA^ gene.

Given the heterogeneity of the eight alleles (Table 1), clear ethnic preponderance of most frequent alleles and the statistical significant difference in the inter-ethnic distribution of most *α* thalassaemia determinants, it could be suggested that an assertion of ethnicity-based hierarchical screening approach for Malaysian multi-ethnic population would be effective in maximising the resources.

### 2.3. Clinical Syndromes Correlated with Haematological Traits

The distribution of four syndromes and their mean haematological values are shown in Table 4 and 5 respectively. Inter-syndrome comparison of haematological values showed a statistically significant difference for haemoglobin, red cells, reticulocyte counts and red blood cell (RBC) indices, including mean corpuscular volume (MCV), mean corpuscular haemoglobin (MCH), mean corpuscular haemoglobin concentration (MCHC) and red cell distribution width (RDW) (analysis of variance (ANOVA), *p* < 0.001). The different fractions of haemoglobins also showed significant quantitative difference amongst the syndromes. The significant differences observed for these dependent variables with ANOVA were further explored by post-hoc Fisher’s least significant difference (LSD) testing. The dependent variables Hb, MCV and MCH showed a significant difference (Fisher’s LSD, *p* < 0.05) in all pairwise phenotypic comparisons. These indicate that Hb, MCV and MCH have important clinical and laboratory repercussions.

The two parameters MCV and MCH may give a significant predictive clue as to the type of *α* thalassaemia carrier in hospital patients. We observed that the mean MCV for silent carriers was 75.8 fL, with a 95% confidence interval (CI) of 75.3–76.4 fL, whilst the measure for both *α*^+^ and *α*^0^ thalassaemia traits were consistently less than 71 fL—*α*^+^ thalassaemia trait measured 69.8 fL (95% CI, 68.6–70.9 fL) and *α*^0^ thalassaemia 67.8 fL (95% CI, 67.4–68.3 fL). Similarly, when the mean MCH for silent carriers was 24.2 pg (95% CI, 24–25.5 pg), the same was at least 1.5 pg lower in 95% cases of *α* thalassaemia traits (21.8 pg, 95% CI, 21.5–22.2 pg for *α*^+^ thalassaemia; 21.2 pg, 95% CI, 21.1–21.4 for *α*^0^ thalassaemia). Therefore, from the observations presented here, these two red cell indices may provide a rough guide for differentiating silent carriers from alpha traits. However, these observations should be interpreted cautiously, as possible co-inheritance of β-thalassaemia and other haemoglobinopathies was not ruled out in these samples.

At antenatal clinics and population screenings, red cell indices such as low MCV and MCH are first line tests suggestive of thalassaemia and haemoglobinopathy. Different schemas have been put forward based on these two indices. The guideline from the British Committee for Standards in Haematology recommends testing for thalassaemia if MCH is less than 27 pg [21]; in Hong Kong, thalassaemia screening is recommended if MCV < 75 fL [22] or < 80 fL [23]; in Sardinia, when MCV < 78 fL and MCH < 27 pg [24]; and for the Chinese population, when MCV < 80 fL and MCH < 27 pg [25]. These rough schemas, however, do not encapsulate all the cases of alpha thalassaemia, and hence may not serve as a reliable diagnostic benchmark in a clinical setting. As was empirically observed here, 422 (19.7%) patients diagnosed with various *α* thalassaemia syndromes in this analysis had an MCV ≥ 80 fL and 109 (4.4%) patients had an MCH ≥ 27 pg. Moreover, 52 patients with *α*^0^ thalassaemia had MCV ≥ 80 fL and 10 patients with this phenotype had MCH ≥ 27 pg. Coexisting β thalassaemia does not, in general, show a salutary effect on MCV and MCH [26]. However, one possible explanation for the masking of microcytosis of thalassaemia may be concomitant folate deficiency [27]. This vitamin deficiency has been reported in thalassaemia because of chronic erythroid hyperplasia. These discordant results from a hospital-based sample suggest that a plausible difference may exist from the population-based red cell indices and a broader differential diagnosis should be sought by ordering other relevant tests beside routine Hb genotype.

The degree of anisocytosis measured in this study by RDW-CV showed a consistent elevation in both *αα*/*αα* genotypes and various syndromes of *α* thalassaemias. This index varied considerably from one form of *α* thalassaemia to another, but did not seem to correlate with the degree of *α* globin deficiency, and hence was ineffective in discriminating the different phenotypes. However, the measure was distinctly elevated in patients with Hb H disease to indicate marked anisocytosis. This increase was possibly due to chronic haemolytic anaemia with polychromatic reticulocytosis with a background of microcytosis, targeting and misshapen red cells. The usefulness of a more recent function, RDW-SD, is yet to be analysed in *α* thalassaemia.

#### 2.3.1. Normal Genotype

Individuals with the *αα*/*αα* genotype presented here showed normal to subnormal levels of haemoglobin and red cell indices, as well as raised RDW and HbA_2_ above the population average. An elevated mean value of HbA_2_ (4.1% ± 7.3%) with concomitant hypochromia and microcytosis could be the result of the presence of β thalassaemia and/or other haemoglobinopathies. As our cases were hospital based, anaemia from chronic disorders and iron deficiency may also be among the differential diagnosis for microcytosis. These potential causes for microcytosis and elevated HbA_2_ were not further investigated.

#### 2.3.2. Alpha Thalassaemia Silent Carriers

Studies have shown that *α* thalassaemia silent carriers (–*α*/*αα*) with deletion are generally indistinguishable from the *αα*/*αα* genotype, as they are essentially normal both clinically and haematologically [1]. The silent carriers in this analysis, however, presented with normal to mild anaemia (Hb 11.5 ± 2.0 g/dL) for their age and sex, subnormal levels of MCV and MCH and a mild elevation in RDW. This was probably because these patients were referred from hospitals, and therefore did not perfectly represent the typical asymptomatic *α* thalassaemia silent carriers in the target population. The most frequent genotypes encountered for our silent carriers were *αα*/*α*–^3.7^ and *αα*/*α*^CS^*α* with a proportion of 3.8:1; this was the case for all ethnic groups. Less frequent were *αα*/–*α*^4.2^ in Malays, Chinese and Indians and the *αα*/*α*^QZ^*α* genotype in Malays and Chinese only. An early diagnosis of –*α*/*αα* can be established in most Malays, Chinese and Indians by demonstrating 1%–2% of Hb Bart’s in the neonatal period [28]. The incidence of silent carriers calculated for our sample set was 20.4%.

#### 2.3.3. Alpha Thalassaemia Trait

Alpha thalassaemia trait is the result of either homozygous *α*^+^ thalassaemia (–*α*/–*α*), heterozygous *α*^0^ thalassaemia (– – /*αα*) or the doubly heterozygous state for *α*^+^ interacting with a nondeletional mutation (–*α*/*αα*^T^). These patients are typically asymptomatic, with mild to moderate microcytic hypochromic anaemia. In general, our analysis showed that *α*^+^ and *α*^0^ thalassaemias were similar in their laboratory presentation, except for the mild depression in haemoglobin and RBC counts in *α*^+^ relative to *α*^0^ thalassaemia trait (Table 5). However, an obvious difference was exhibited in a slight elevation in Hb F per cent (2.9% ± 13.1%) in homozygous *α*^+^ thalassaemia beyond the age of one year.

#### 2.3.4. Hb H Disease

Hb H disease has a multiallelic basis. This syndrome in Malaysia, like in many other Southeast Asian and Mediterranean populations, was most commonly (66.4%) caused by deletion of three genes (– – /–*α*). Double heterozygosity for *α*^0^ thalassaemia and a nondeletional mutant (– – /*α*^T^*α*) accounted for 33.6% of the syndrome. Consistent with previous studies [29], Constant Spring was the most common nondeletional mutation (61/375, 16.3%) causing nondeletional Hb HCS.

Hb H disease showed a clear distinction in mean MCHC, RDW and reticulocyte count from the rest of the phenotypes (Fisher’s LSD, *p* < 0.05). Erythrocyte counts between Hb H disease (mean RBC count = 4.7 ± 0.99) and normal genotype (mean RBC count = 4.6 ± 0.76), and between Hb H disease and silent carriers (mean RBC count = 4.7 ± 0.72) showed no significant difference (Fisher’s LSD, *p* > 0.05) in their distributions. Low average HbA^2^ values (2.4 ± 2.4%) observed in Hb H disease were significantly different (Fisher’s LSD, *p* < 0.05) from the population average high values observed in normal (4.1% ± 7.3%) and silent carriers (3.7 ± 6.4%). Laboratory features of Hb H disease typically showed hypochromic microcytic anaemia (8.8 ± 1.4 g/dL, MCV 64.9 ± 12.6 fL) compensated by reticulocytosis (4.3% ± 5.2%) and marked anisocytosis (RDW 26.2 ± 6.7). Mild elevation in HbF was also seen.

While analysing the data for this study, we encountered 22 patients who had compound heterozygotes for nondeletional variant *α*^CD59^*α*/*α*^CS^*α* clinically presenting as severe Hb H–like disease. Out of these, 21 were ethnic Malays and a single case came from the Orang Asli group. We noted with interest that these patients presented with significant anaemia at an earlier age—one patient as early as 6 weeks of age—while the rest mostly presented with anaemia at 2–3 years of age, and required more frequent blood transfusions. It was also noted that 56 patients were diagnosed as compound heterozygous with the *α*^–3.7^/*α*^CD59^*α* genotype. Some of these patients presented as thalassaemia intermedia with moderate anaemia. It is also interesting to note that no case of Hb Adana was observed to be co-inherited with *α*^0^ thalassaemia in this analysis. This is perhaps further evidence that the combination is nearly always a lethal condition. The clinical presentations observed here in our patients are highly suggestive that Hb Adana should be considered an *α*^0^ phenotype, unless further analysis to determine the phenotype-genotype correlation indicates otherwise. The relatively high frequency of the *α*^CD59^ mutation observed in the Malay ethnic group along with their increased array of *α* thalassaemia determinants should alert relevant health personnel of the possibility of severe phenotypes arising from symptomless alpha thalassaemia trait parents through compound heterozygosity with *α*^CD59^. It may also be useful to investigate for codon 59 mutation in the *α*2 globin gene in Malay women who experience miscarriages.

## 3. Experimental Section

IMR serves as the national reference centre for diagnostic molecular genetic diseases. Clinical and laboratory data from 5016 unrelated patients referred to IMR for *α* thalassaemia testing from various geographically distinct hospitals between 2007 and 2010 were retrieved. Laboratory data accessed included complete blood counts (CBC), Hb chromatogram findings and *α* globin genotyping results done at IMR. To preserve the anonymity of these patients, demographic data accessed were limited to ethnicity only.

In most cases, initial blood work was performed at the referring hospital laboratory and included CBC counts from automated haematology analysers and Hb chromatography analysis from Bio-Rad Variant or Variant II high performance liquid chromatography (Bio-Rad Laboratories, Hercules, CA, USA). Blood specimens were transported in K_3_EDTA tubes at 4 °C to IMR from the referring hospitals along with the requisition form and initial blood investigation results. However, those blood samples referred without CBC and Hb chromatogram information were tested at IMR on an automated blood analyser (CellDyn 3200, Abbott Laboratories, IL, USA) and a VARIANT Haemoglobin Testing System (Bio-Rad Laboratories, Hercules, CA, USA), respectively.

For molecular studies, genomic DNA was extracted from whole blood using the standard protocol of Qiagen Inc. (Valencia, CA, USA). Alpha thalassaemia screening panel consisted of 13 deletions and mutations representing common *α* thalassaemia determinants encountered in South-East Asia, India and Middle East. The single-tube seven-plex PCR method was applied to detect the two most common single gene deletions *α*–^3.7^ and –*α*^4.2^, and double gene deletions including – – ^SEA^, – – ^FIL^, – – ^THAI^ and ––^MED^ and –(*α*)^20.5^, as previously described [30]. For the detection of *α*2 globin gene mutations, the single-tube multiplex amplification refractory mutation system (ARMS) method was used as previously described [31]. The nondeletional mutations detected by this method were *α*^CS^*α*, the codon 125 CTG→CCG mutation or Hb Quong Sze, the codon 59 (GGC→GAC) mutation, the initiation codon mutation (ATG→A–G), the codon 30 mutation (ΔGAC) and the codon 35 mutation (TCC→CCC). All the amplification reactions were done on an Eppendorf Mastercycler^®^ (Eppendorf Scientific, Hamburg, Germany). We also used the *α*-thalassaemia StripAssay^®^ hybridisation test commercially available from ViennaLab Diagnostics GmbH, Vienna, Austria for cases where the nondeletional mutations were suspected to exhibit the homozygous state. All subsequent diagnoses of *α* thalassaemia status were reported to the respective referring centres.

Statistical analyses were performed using ANOVA to compare parametric means of haematological variables measured within the *α* thalassaemia phenotypic groups, and post-hoc range tests of LSD were used to determine which parametric means differed by pair-wise comparison of different phenotypes. Difference in the incidence of each allele among ethnic groups was checked using the Pearson Chi-square test. The associations amongst ethnic group and *α* genotype frequencies were evaluated using the non-parametric Kruskal-Wallis test. The two-sample non-parametric Mann-Witney *U* test (at *p* < 0.05) was employed to check the null hypothesis that any two ethnic groups had the same distribution of *α* thalassaemia phenotypes. All data were analysed using IBM SPSS software version 20 (IBM Corp., Armonk, NY, USA).

Ethical approval for this project was obtained from the ethics committee of the Ministry of Health Malaysia.

## 4. Conclusions

We described the molecular epidemiological characteristics of *α* thalassaemia in Malaysia. Significant differences in its prevalence, molecular defects and phenotypic expression exist amongst different geographic locations and ethnic groups. We observed a genetic heterogeneity of the common alpha thalassaemia alleles in all major ethnic groups. Hence, the data suggest that an ethnicity-based hierarchical screening protocol should be implemented to screen *α* thalassaemia determinants in relation to the frequency of alleles in each subpopulation.

The prevalence data on the plethora of *α* thalassaemia determinants observed in this study may help in estimating the disease heterogeneity amongst the various ethnic groups of Malaysia. Since our study sample comprised a wide representation of multiple ethnic groups of the peninsula and west Malaysia, the findings could safely be generalised to the target subpopulations and should prove useful in carrier screening programmes, genetic counselling and patient management.

## Figures and Tables

**Table 1:** Alpha thalassaemia alleles and their relative incidences in different ethnic groups.

Ethnic group	Alpha gene alleles								

	*αα*	*α*–^3.7^	––^SEA^	*α*^CS^*α*	Cd59 G > A	–*α*^4.2^	––^FIL^	––^THAI^	*α*^QZ^*α*
Malay, *n* (%)	4353 (69.3%)	840 (13.4%)	581 (9.2%)	273 (4.3%)	113 (1.8%)	83 (1.3%)	9 (0.14%)	18 (0.28%)	8 (0.13%)
Chinese, *n* (%)	854 (60.5%)	88 (6.2%)	423 (29.9%)	10 (0.70%)	2 (0.14%)	16 (1.1%)	0	6 (0.42%)	13 (0.92%)
Indian, *n* (%)	495 (86.8%)	60 (10.5%)	7 (1.2%)	0	0	6 (1.0%)	1 (0.2%)	1 (0.2%)	0
Sabah, *n* (%)	470 (60.1%)	227 (29.0%)	67 (8.6%)	10 (1.3%)	2 (0.25%)	0	6 (0.76%)	0	0
Sarawak, *n* (%)	56 (68.3%)	11 (13.4%)	2 (2.4%)	0	3 (3.7%)	0	10 (12.2%)	0	0
Orang Asli, *n* (%)	33 (63.5%)	7 (13.5%)	5 (9.6%)	6 (11.5%)	1 (1.9%)	0	0	0	0
*p* value		<0.001	<0.001	<0.001	<0.001	0.792	<0.001	0.574	<0.001
Ethnicity unknown, n (%)	633 (73.9%)	105 (12.3%)	80 (9.3%)	17 (2.0%)	9 (1.0%)	6 (0.7%)	1 (0.1%)	1 (0.1%)	4 (0.5%)
Total number of chromosomes	6894 (68.7%)	1338 (13.3%)	1165 (11.6%)	316 (3.2%)	130 (1.30%)	111 (1.1%)	27 (0.27%)	26 (0.26%)	25 (0.25%)

**Table 2:** Genotype frequencies of the alpha-globin gene cluster in different ethnic groups.

Genotypes	Ethnicity							Total

	Malay	Chinese	Indian	Sabah	Sarawak	Orang Asli	Unknown	
*αα*/*αα*	1571 (50.0%)	220 (31.2%)	217 (76.1%)	165 (42.2%)	22 (53.7%)	12 (46.2%)	242 (56.5%)	2449 (48.8%)
*αα*/––^SEA^	423 (13.5%)	360 (51.0%)	6 (2.1%)	33 (8.4%)	1 (2.4%)	2 (7.7%)	57 (13.3%)	882 (17.6%)
*αα*/*α*–^3.7^	486 (15.5%)	31 (4.4%)	48 (16.8%)	100 (25.6%)	4 (9.8%)	5 (19.2%)	73 (17.1%)	747 (14.9%)
*α*–^37^/– –^SEA^	120 (3.8%)	45 (6.4%)	1 (0.4%)	32 (8.2%)	1 (2.4%)	1 (3.8%)	14 (3.3%)	214 (4.27%)
*αα*/*α*^CS^*α*	175 (5.6%)	5 (0.7%)	0	4 (1.0%)	0	2 (7.7%)	8 (1.9%)	194 (3.87%)
*α*–^3.7^/*α*–^3.7^	68 (2.2%)	4 (0.6%)	5 (1.8%)	44 (11.3%)	0	0	5 (1.2%)	126 (2.51%)
*αα*/–*α*^4.2^	53 (1.7%)	5 (0.7%)	5 (1.8%)	0	0	0	3 (0.7%)	66 (1.32%)
*α*–^3.7^/*α*^Cd59^*α*	45 (1.4%)	1 (0.1%)	0	2 (0.5%)	3 (7.3%)	0	5 (1.2%)	56 (1.12%)
*αα*/*α*^Cd59^*α*	44 (1.4%)	1 (0.1%)	0	0	0	0	4 (0.9%)	49 (0.98%)
*α*–^3.7^/*α*^CS^*α*	41 (1.3%)	1 (0.1%)	0	3 (0.8%)	0	1 (3.8%)	2 (0.5%)	48 (0.96%)
––^SEA^/*α*^CS^*α*	24 (0.8%)	4 (0.6%)	0	2 (0.5%)	0	2 (7.7%)	6 (1.4%)	38 (0.76%)
–*α*^4.2^/––^SEA^	14 (0.4%)	10 (1.4%)	0	0	0	0	2 (0.5%)	26 (0.52%)
*α*^Cd59^*α*/*α*^CS^*α*	21 (0.7%)	0	0	0	0	1 (3.8%)	0	22 (0.44%)
*αα*/––^THAI^	16 (0.5%)	4 (0.6%)	1 (0.4%)	0	0	0	1 (0.2%)	22 (0.44%)
*αα*/––^FIL^	9 (0.3%)	0 (0.42%)	1 (0.4%)	3 (0.8%)	7 (17.1%)	0	0	20 (0.40%)
*αα*/*α*^QZ^*α*	5 (0.2%)	8 (1.1%)	0	0	0	0	3 (0.7%)	16 (0.32%)
*α*–^3.7^/–*α*^4.2^	9 (0.3%)	1 (0.1%)	1 (0.4%)	0	0	0	0	11 (0.22%)
*α*–^3.7^/– –^FIL^	0	0	0	2 (0.5%)	3 (7.3%)	0	1 (0.2%)	6 (0.12%)
––^SEA^/*α*^QZ^*α*	0	4 (0.6%)	0	0	0	0	1 (0.2%)	5 (0.10%)
–*α*^4.2^/*α*^CS^*α*	3 (0.1%)	0	0	0	0	0	1 (0.2%)	4 (0.08%)
*α*^CS^*α*/*α*^CS^*α*	4 (0.1%)	0	0	0	0	0	0	4 (0.08%)
*α*–^3.7^/– –^SEA^	2 (0.1%)	1 (0.1%)	0	0	0	0	0	3 (0.06%)
–*α*^4.2^/*α*^Cd59^*α*	3 (0.1%)	0	0	0	0	0	0	3 (0.06%)
––^FIL^/*α*^CS^*α*	0	0	0	1 (0.3%)	0	0	0	1 (0.02%)
––^THAI^/*α*^QZ^*α*	0	1 (0.1%)	0	0	0	0	0	1 (0.02%)
*α*–^3.7^/*α*^QZ^*α*	1 (0.03%)	0	0	0	0	0	0	1 (0.02%)
–*α*^4.2^/*α*^QZ^*α*	1 (0.03%)	0	0	0	0	0	0	1 (0.02%)
*α*^QZ^*α*/*α*^CS^*α*	1 (0.03%)	0	0	0	0	0	0	1 (0.02%)
**Total**	**3139**	**706**	**285**	**391**	**41**	**26**	**428**	**5016 (100%)**

**Table 3:** Ethnic distribution of normal and alpha thalassaemia phenotypes.

Ethnicity		Alpha thalassaemia syndromes			
	
	(*αα*/*αα*)	Silent carrier	*α*^+^ thal trait	*α*^0^ thal trait	Hb H disease
Malay	1571 (50.0%)	719 (22.9%)	128 (4.1%)	492 (15.7%)	229 (7.3%)
Chinese	220 (31.2%)	49 (6.9%)	6 (0.84%)	365 (51.7%)	66 (9.3%)
Indian	217 (76.1%)	53 (18.6%)	6 (2.1%)	8 (2.8%)	1 (0.4%)
Sabah	165 (42.2%)	104 (26.6%)	47 (12.0%)	36 (9.2%)	39 (9.9%)
Sarawak	22 (53.6%)	4 (9.7%)	0	8 (19.5%)	7 (17.1%)
Orang Asli	12 (53.8%)	7 (11.5%)	1 (7.7%)	2 (23.1%)	4 (3.8%)
Unknown	242 (59.3%)	87 (12.6%)	8 (4.4%)	62 (21.7%)	29 (1.9%)

**Table 4:** Pairwise analysis of Mann-Whitney *U* test statistics for genotype distributions among different ethnic groups.

	Mann-Whitney *U* test statistic *p*-values					
	
	Malay	Chinese	Indian	Sabah	Sarawak	Orang Asli
**Malay**	−	<0.001	<0.001	0.080	0.510	0.642
**Chinese**		−	<0.001	<0.001	0.328	0.209
**Indian**			−	<0.001	<0.001	<0.001
**Sabah**				−	0.712	0.989
**Sarawak**					−	0.983
**Orang Asli**						−

**Table 5:** Haematological parameters for normal and alpha thalassaemia phenotypes.

	Phenotype					
	
Measured parameters	(*αα*/*αα*)	Silent carrier	*α*^+^ thal trait	*α*^0^ thal trait	Hb H disease	*p* value
Age (years), mean ± SD	20.6 ± 15.5	21.0 ± 15.7	21.3 ± 16.5	20.7 ± 15.2	21.7 ± 15.3	0.860
Hb (g/dL), mean ± SD	11.1 ± 2.2	11.5 ± 2.0	10.8 ± 1.7	11.3 ± 1.7	8.8 ± 1.4	<0.001
RBC (mil/μL), mean ± SD	4.6 ± 0.76	4.78 ± 0.72	4.97 ± 0.81	5.32 ± 0.81	4.72 ± 0.99	0.000
RDW (%), mean ± SD	19.7 ± 8.2	17.9 ± 6.1	17.8 ± 5.4	18.0 ± 4.9	26.2 ± 6.7	<0.001
MCV (fL), mean ± SD	75.1 ± 10.0	75.8 ± 8.5	69.8 ± 8.1	67.8 ± 7.2	64.9 ± 12.6	<0.001
MCH (pg), mean ± SD	23.9 ± 3.8	24.2 ± 3.6	21.8 ± 2.4	21.2 ± 2.1	19.1 ± 3.2	<0.001
MCHC (g/dL), mean ± SD	31.8 ± 5.1	31.8 ± 2.2	31.4 ± 2.2	31.5 ± 3.1	30.4 ± 3.0	<0.001
Hb A (%), mean ± SD	84.1 ± 12.6	83.5 ± 15.6	85.2 ± 11.3	86.1 ± 6.1	86.0 ± 12.5	0.048
Hb A_2_ (%), mean ± SD	4.1 ± 7.3	3.7 ± 6.4	3.4 ± 3.5	3.1 ± 4.3	2.4 ± 2.4	0.002
Hb F (%), mean ± SD	2.7 ± 10.6	1.8 ± 7.8	2.9 ± 13.1	0.9 ± 2.6	2.0 ± 4.9	0.003
Retic (%), mean ± SD	1.7 ± 3.0	1.9 ± 4.5	1.9 ± 1.5	1.3 ± 1.6	4.3 ± 5.2	0.000
